# Photodegradation of Stearic Acid Adsorbed on Superhydrophilic TiO_2_ Surface: In Situ FT-IR and LDI Study

**DOI:** 10.1186/s11671-015-1210-y

**Published:** 2015-12-29

**Authors:** Natalia Smirnova, Tatiana Fesenko, Maxim Zhukovsky, Jacek Goworek, Anna Eremenko

**Affiliations:** O.Chuiko Institute of Surface Chemistry, Ukrainian National Academy of Sciences, 17 General Naumov, Kyiv, 03680 Ukraine; Faculty of Chemistry, Maria Curie-Skłodowska University, Pl. Maria Curie-Skłodowskiej 3, 2-031 Lublin, Poland

**Keywords:** Mesoporous TiO_2_ films, Photo-induced superhydrophilicity, FT-IR, Laser desorption–ionization mass spectroscopy, Stearic acid photodegradation, 81.15.Gh, 82.33Ya

## Abstract

TiO_2_ films prepared by template-assisted sol–gel method were characterized by X-ray diffraction spectroscopy, scanning and atomic force electron microscopy, and Fourier transform infrared (FT-IR) spectroscopy. Based on the hexane adsorption–desorption analysis, the films have a surface area of 390–540 m^2^/g with pore size distribution narrowly centered around 10 nm. Optimal component ratio and condition of heat treatment of mesoporous titania films have been found. Photocatalytic activity of the coatings was determined by the destruction of stearic acid layers, monitored using FT-IR spectroscopy and laser desorption–ionization (LDI) mass spectrometry. Under UV illumination, all the used films reach hydrophilicity with water contact angle of 0°. As the result, hydrophobic fat acid molecules undergo self-association and active desorption from the hydrophilic surface during mass-spectrometric experiment.

## Background

The decomposition of organic molecules using TiO_2_ as a photocatalyst is well known [1–3]. However, the less attended but equally important fields of TiO_2_ application (air and water pollution control [4, 5], antifogging, self-cleaning glasses production, and so on) are based on photoinduced hydrophilicity [6–8] of this material. This property, along with its semiconductor photocatalytic activity, is maintained by the UV component of sunlight so that any contaminant on the surface will be either photomineralized or readily washed away by rain water. As it was concluded from the results of XPS, Fourier transform infrared (FT-IR), and electrochemical experiments [8, 9], the increase in the hydrophilicity can be attributed to the increase in the number of surface hydroxyl groups on TiO_2_ surface, which are formed by the involvement of the photogenerated holes. Various methods have been proposed to enhance the hydrophilic conversion property such as heat treatment; UV, plasma, or non-linear irradiation [10]; and surface fluorination [11]. Some authors report superhydrophilic properties of nanocomposite TiO_2_/SiO_2_, TiO_2_/WO_3_, and TiO_2_/ZnO films [12–15].

The generally preferred method for assessing the activity of a self-cleaning titania photocatalyst film is the stearic acid (SA) test, in which a thin layer of SA is deposited onto the film and its photocatalytic destruction is monitored as a function of time [16–18]. The photocatalytic destruction of SA is of practical interest since it provides a reasonable model compound for the type of solid organic films that pollute different glass surfaces, such as a kitchen window or a light cover in a road tunnel. In the presented work, the variation in SA level and intermediate products of this process was monitored as a function of irradiation time using FT-IR and laser desorption–ionization (LDI) mass spectrometry investigation.

Recently, some nanomaterials (TiO_2_, ZnO, Fe_3_O_4_/TiO_2_ сore/shell, TiO_2_/Ag nanoparticles) have been used to assist LDI of biomolecules for mass spectrometry analysis. Comparing with classical matrix-assisted LDI, strategies based on nanomaterial-assisted ionization minimize background peaks, which is of great benefit for the qualitative and quantitative analysis of small biomolecules [19–22]. TiO_2_ is a semiconductor with a large bandgap (bulk anatase: 3.2 eV) and can therefore be used as a SALDI matrix with the N_2_ laser (337 nm). Chen et al. succeeded in the ionization of low molecular weight compounds, peptides, and oligosaccharides with sol–gel TiO_2_ films [23–26]. Our previous studies [23, 24, 27] have demonstrated the feasibility of mesoporous sol–gel films as the substrate for matrix-free LDI-MS analysis that gives us a possibility for in situ investigation of phototransformations of adsorbed dye.

In this work, we focused on the synthesis, characterization, and assessment of the self-cleaning properties of photoactive mesoporous TiO_2_ coatings. Photodegradation of SA has been used as a model reaction as the fatty acids contribute to the greasy contaminations of outdoor architecture elements. Effect of treatment temperature on photocatalytic activity of TiO_2_ films and mechanism of SA photodegradation has been studied via combination of in situ FT-IR and LDI spectroscopies.

## Methods

Titanium (IV) tetraisopropoxide (TTIP), three-block copolymer of polyethylene oxide and polypropylene oxide (PEO)_20_(PPO)_70_(PEO)_20_ (Pluronic P123), acetylacetone, concentrated hydrochloric acid (HCl (36.6 wt.%)), and SA of reagent grade purity were purchased from Aldrich and used without additional purification.

Nanocrystalline TiO_2_ films on glass and steel substrates were prepared by sol–gel method similarly to [25], using TTIP, three-block copolymer Pluronic P123 as a template, acetylacetone as a complex agent, and hydrochloric acid as a sol–gel transition catalyst. The molar ratios of the components in the sol for film deposition were as follows: TTIP:P123:acetylacetone:HCl:H_2_O:C_2_H_5_OH = 1:0.05:0.5:4.6:10:41. The primary films were deposited onto glass or stainless steel plates from a colloidal solution by the “dip-coating” method and dried at 100 °C. The dried films were calcined at 350, 400, or 500 °C in air.

X-ray diffraction (XRD) patterns of the films were recorded using Cu Kα radiation on a DRON-4-07 in the 2*θ* range from 10 to 60°.

The surface morphology of the films was observed using scanning electron microscopy (SEM) on a LEO 1530 Scanning Electron Microscope, and atomic force microscopic (AFM) images for the films were obtained from a Nanoscope IIIa setup.

The reflection spectra of films in the 400–2000 cm^−1^ range have been measured using a FT-IR (Thermo Nicolet NEXUS) spectrophotometer.

The Brunauer–Emmett–Teller (BET) surface areas and pore size distributions of the thin films coated onto the covering slides for microscopy examination were obtained from their hexane adsorption/desorption isotherms.

The hydrophilic property was evaluated by examining the contact angle for water on the surface of various TiO_2_ thin films. Assuming that the geometry of the drop is a spherical section, the contact angle can be estimated directly from the diameter of the contact circle measured by an optical microscope. Contact angle *θ* was calculated by the equation *θ* = 2 tan^−1^(*h*/*r*) from the radius *r* and the height *h* of the water drop observed by optical microscopy (SMZ-U, Nikon Corporation).

Photocatalytic behavior was measured under UV radiation. The degradation of SA was followed by FT-IR (Bruker, Vector 22) and LDI spectroscopy. Stearic acid was deposited onto the surface of TiO_2_ films from 20 g/l methanol solution by the dip-coating technique with a constant withdrawal speed of 1.5 mm/s. The activity of the film was defined in cm^−1^ min^−1^, which indicated the rate of reduction in selected SA peaks in the IR region. Under LDI mass spectrometry experiments, the positive and negative ion mass spectra were acquired using an Autoflex II (Bruker Daltonics Inc., Germany) mass spectrometer. The samples were irradiated with a 337-nm nitrogen laser operated at 20 Hz (3 ns pulse duration) and attenuated with neutral density filter. A delayed extraction period of 10 ns was used to minimize the energy spread of the ions for optimum resolution; then, the ions were accelerated by 20-kV pulse through a reflectron time-of-flight analyzer and detected using a multichannel plate detector [23, 27].

## Results and Discussion

**Fig. 1:**
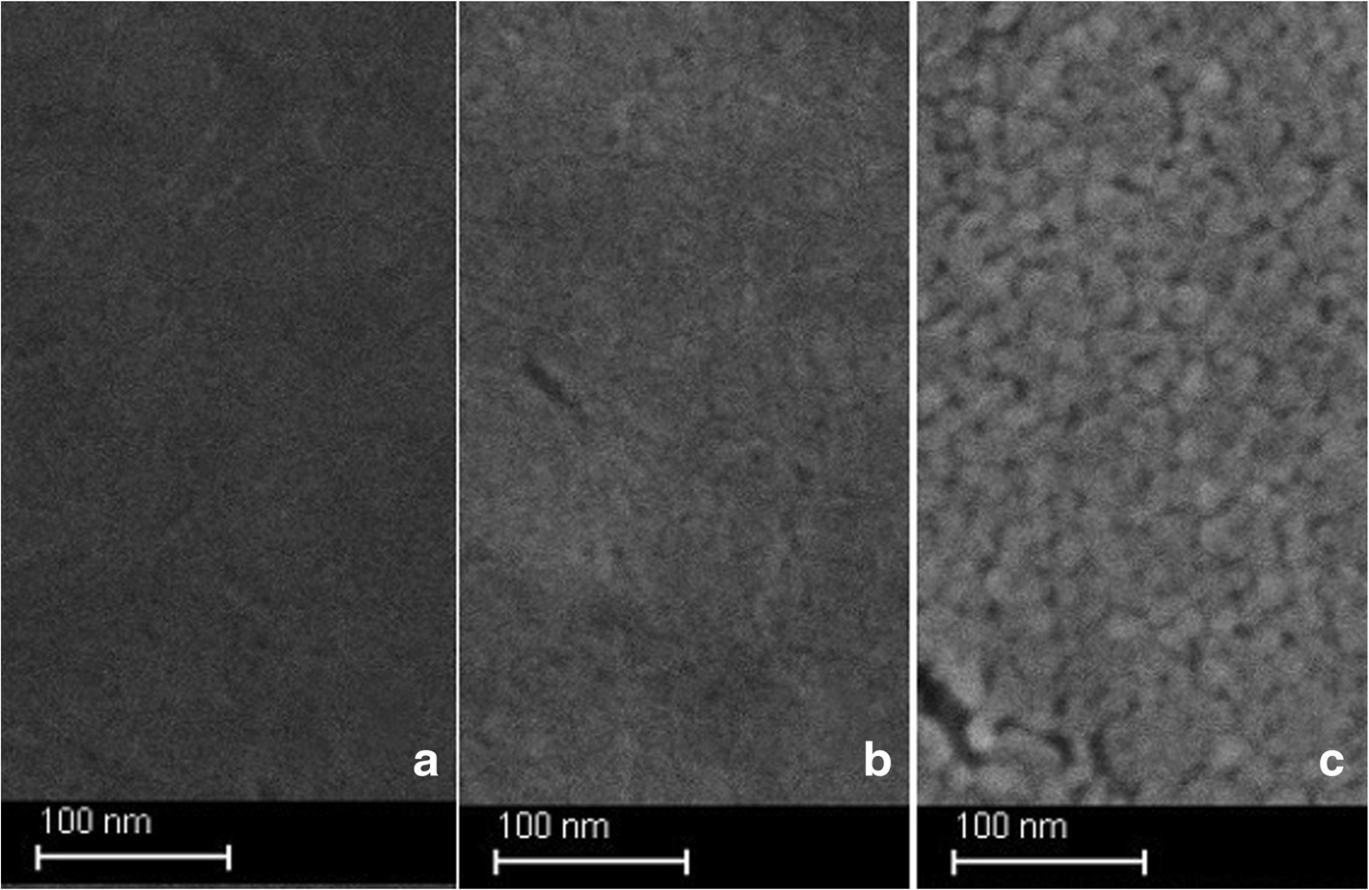
SEM images of mesoporous TiO_2_ thin films calcined at 300 °C (**а**), 400 °C (**b**), and 500 °C (**c**)

**Fig. 2:**
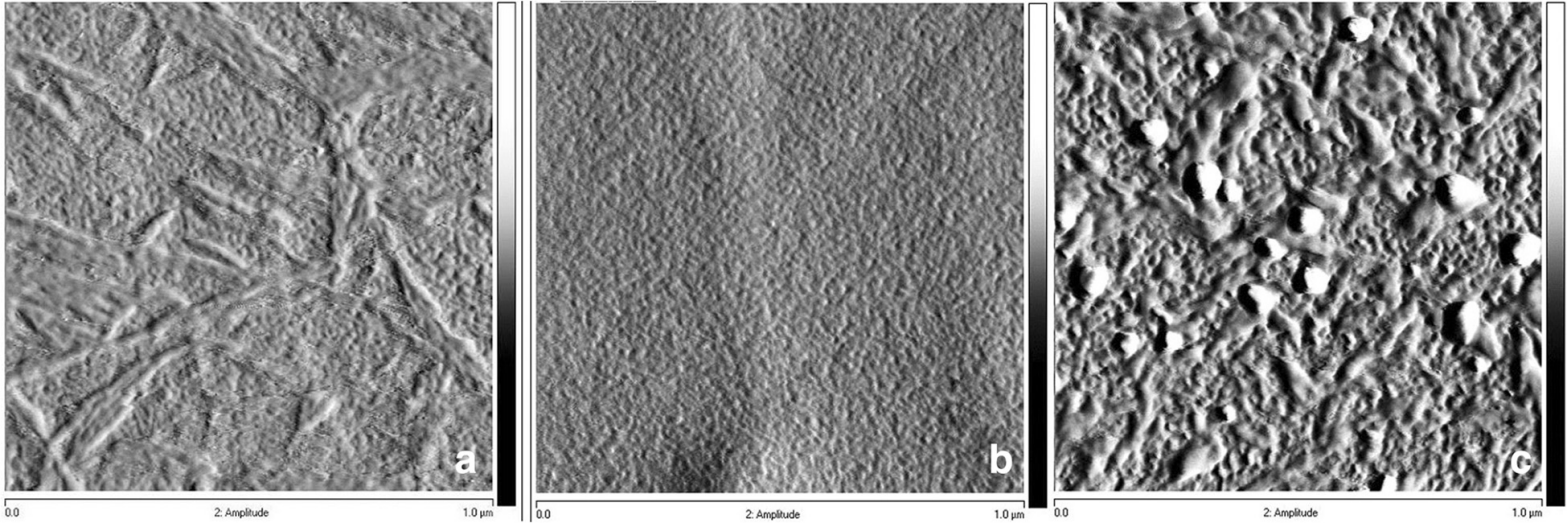
AFM images for the surface of TiO_2_ films. **a** TiO_2_ (350°С). **b** ТіО_2_ (400°С). **c** ТіО_2_ (500°С)

The morphology of the as-prepared films was investigated by SEM and AFM techniques as shown in Figs. 1 and 2. The SEM image reveals agglomeration of near spherical shape TiO_2_ particles. The surface of this film obviously becomes rough due to non-close packing of particles with shapes having corners and edges. The surface phenomenon would be effective to higher depth as compared with smoother films. Further evidences for quantitative measurements of surface roughness of mesoporous TiO_2_ films with heat treatment (350–500 °C) were analyzed by AFM (Fig. 2). The same trend was observed as shown by SEM images.

The uniform surface of TiO_2_ film calcined at 350 °C has developed porous structure with mean pore size about 10 nm. The surface relief of TiO_2_ films calcined at 400 and 500 °C becomes more complex, revealing titanium dioxide crystallization tendency with TiO_2_ aggregates clearly visible on the film surface heat treated at the highest temperature. The last is reflected in the changes of surface roughness *Rq* values from 1.2 through 1.4 up to 4.0 nm for the films calcined at 350, 400, and 500 °C, respectively.

As seen from the presented results (Table 1), all samples are characterized by a developed surface. Obviously through structure-directing action of template is achieved relatively orderly organization of titanium dioxide crystallites formed on the surface of the glass substrate during deposition and subsequent heat treatment. The increase of heat treatment temperature from 350 to 400 °C leads to growth of the specific surface area of prepared films as the result of a deeper burnout of organic components of the gel. Further reduction in *S*_BET_ values after heat treatment at higher temperature (500 °C) is a sign of a crystallization process that promotes the conglomeration of primary aggregates of titanium dioxide and further consolidation and improvement of its spatial packing. The crystallization and sintering of TiO_2_ films at high temperatures, resulting, on the one hand, in the partial destruction of porous structure, on the other hand, can contribute to enlargement of pores due to their association that probably leads to a decrease in *S*_BET_ of calcined samples.

**Table 1:** Specific surface area *S*_BET_ for mesoporous TiO_2_ films derived from hexane adsorption–desorption isotherms and their wettability

Temperature of thermal treatment of TiO_2_ film	350 ^о^С	400 ^о^С	500^ о^С	15 min of UV irradiation
*S*_BЕТ_ (m^2^/g)	495	542	392	
Water contact angle (grad ^o^)	23	17	< 10	0

**Fig. 3:**
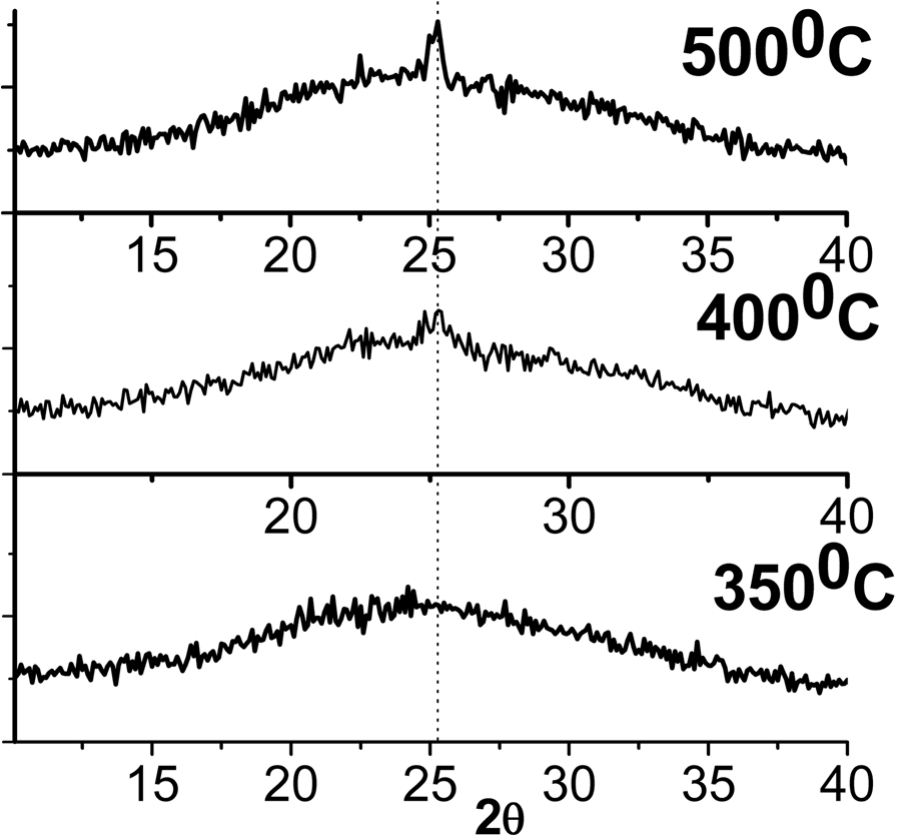
XRD pattern of the TiO_2_ films after thermal treatment at 350, 400, and 500 °C

Figure 3 illustrates the XRD patterns of the TiO_2_ thin films obtained after eight dip-coating procedures and various annealing temperatures at 350, 400, and 500 °C. The feature common to all the XRD patterns is a broad band in the low 2*θ* region of 15 to 35, which characterizes an amorphous phase or glass substrate. Anatase phase has been identified in XRD pattern for film calcined at 500 °C.

### Hydrophilic Properties

The available literature data concerning the contact angle of water on a clean titania surface are quite contradictory, ranging from 72° reported in [28] to 33.3° in [28] and 15° in [29] depending on the film structure and storage conditions. According to Hashimoto et al., the wettability of TiO_2_ surfaces can be altered by the irradiation [28–30].

Hydrophilic properties of the TiO_2_ films were estimated from measurements of the water contact angle (see data listed in Table 1). Increasing of the heat treatment temperature of the TiO_2_ films from 350 to 500 °C was accompanied by the decrease of the water contact angle of the samples from 23 to 10°, indicating their improved natural hydrophilicity. This could be ascribed to the higher degree of residual organics burning out in the templated TiO_2_ sol–gel films. On the other hand, as the surface hydrophilicity could be correlated with the thermal treatment-induced surface defects of TiO_2_, it is possible to assume that the decrease of the water contact angle for the titania films calcined at higher temperatures is a result of formation of more defect structure. Desorption of surface oxygen at high temperatures produces oxygen vacancies and Ti^3+^ sites, which could be occupied by water molecules producing OH groups rendering the surface more hydrophilic in character. After UV illumination (15 min in air under mercury lamp light), the mesoporous TiO_2_ films exhibited photoinduced superhydrophilicity with water contact angle near 0°. Various experimental results relating to this phenomenon have shown that it is caused by defects formed on the surfaces of the as-prepared TiO_2_ thin films subjected to high-temperature calcination, especially in case of template syntheses involving high level of organic component as it was used in our work.

Reducing of contact angle with increasing of the temperature (Table 1) of previous heat treatment is consistent with the results of thermal analysis [26]. Basic loss of weight for titanium dioxide powder obtained via sol–gel transformation of relevant films precursor is observed in the temperature range from 200 to 340 °C and coincides with template removal. A slight weight loss in temperature interval 340–450 °C can be attributed to the decomposition of residual acetylacetonate groups containing carbon.

**Fig. 4:**
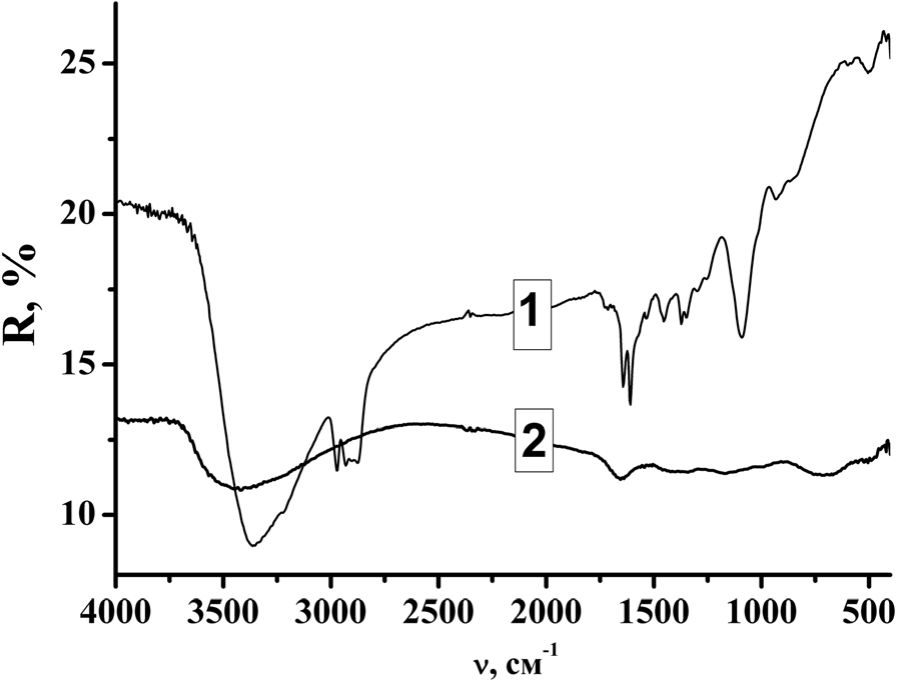
FT-IR spectra of the TiO_2_ films deposited onto steel substrates as prepared (*1*) and after thermal treatment at 500 °C (*2*)

The IR spectroscopy with Fourier transformation gives us additional information about the film surface. In the IR spectra of as-prepared TiO_2_ films (Fig. 4), intense broad band near 3300–3400 cm^−1^ characteristic of the Ti–OH vibrations was observed. Three peaks at 2980–2870 cm^−1^ can be attributed to vibrations in C–H bonds of organic components. Peaks within 1650–1530 cm^−1^ indicate the formation of bidentate complex with keto–enol tautomery formed by acetylacetone and titanium isopropoxide [31].

The IR spectra of mesoporous TiO_2_ films after heat treatment (Fig. 4) confirmed burnout of organic components of the gel sample after annealing and a high degree of surface hydroxylation after calcination at 500 °C that indicated by the presence of bands corresponding to the stretching vibrations in 3400 cm^−1^ (Ti–OH) and 1640 cm^−1^ (H–OH) characteristic of the OH groups adsorbed on the TiO_2_ surface. Furthermore, peaks in the 960–400 cm^−1^ are characteristic vibrational modes for TiO_2_ (Ti–O) [32].

### Stearic Acid Photodegradation

Degradation of SA on the surface of mesoporous TiO_2_ films under UV light irradiation depending on the film calcination temperature has been studied in this work in order to elucidate the influence of structural parameters onto the self-cleaning properties of mesoporous titania films.

**Fig 5:**
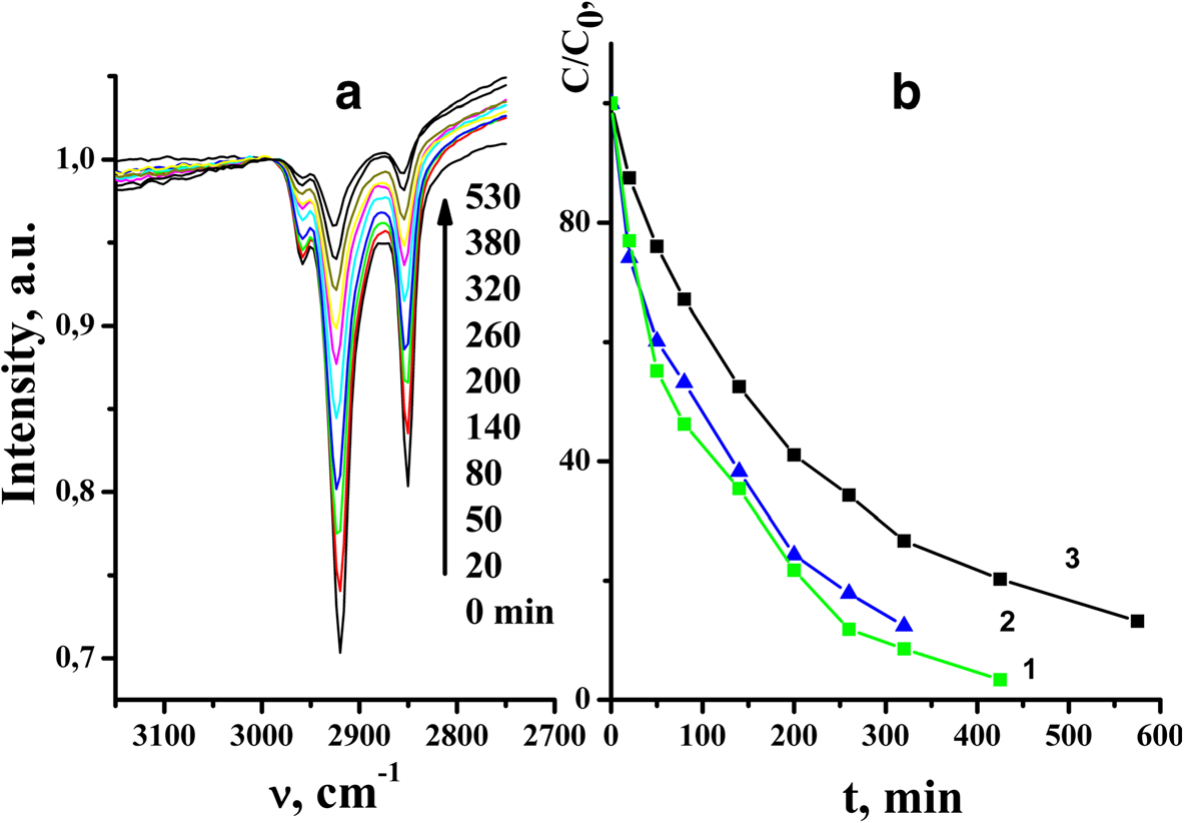
**а** FT-IR spectra of stearic acid on the surface of mesoporous TiO_2_ films. **b** Stearic acid photodegradation on the surface of ТіО_2_ 350°C (*1*), 400°C (*2*), and 500^о^С (*3*) under UV light irradiation

Stearic acid was deposited onto the surface of TiO_2_ films from 20 g/l methanol solution of SA by dip-coating technique with a constant withdrawal speed of 1.5 mm/s. The presence of SA on the surface of TiO_2_ films has been monitored by FT-IR spectroscopy in the range of asymmetric and symmetric stretching vibrations of C–H bonds of CH_2_ groups at 3100–2750 cm^−1^ (Fig. 5).

We have observed that the intensity of *ν*_as, s C–H_ of SA on the surface of mesoporous TiO_2_ films differs significantly depending on the heat treatment temperature of the films. The highest deposition of SA was observed for the films heat treated at 350 °C and the lowest for those calcined at 500 °C. There are several factors that might influence the deposition of SA: specific surface area (surface structural properties) and hydrophilic/hydrophobic properties of the films. Taking into account the results on water contact angle for the studied films, we can state that the surface of mesoporous TiO_2_ films is more hydrophobic after calcination at 350 °C than that of the films heat treated at 400 and 500 °C. As it follows from the conventional concept on wetting that hydrophobicity of a surface results from the uniform distribution of hydrophobic groups [8] (in our case, partially degraded organics), this leads to better affinity between surface and nonpolar parts of SA molecules and results in a higher deposition degree of the fatty acid. Products of SA degradation were identified by LDI mass-spectrometric measurements.

**Fig. 6:**
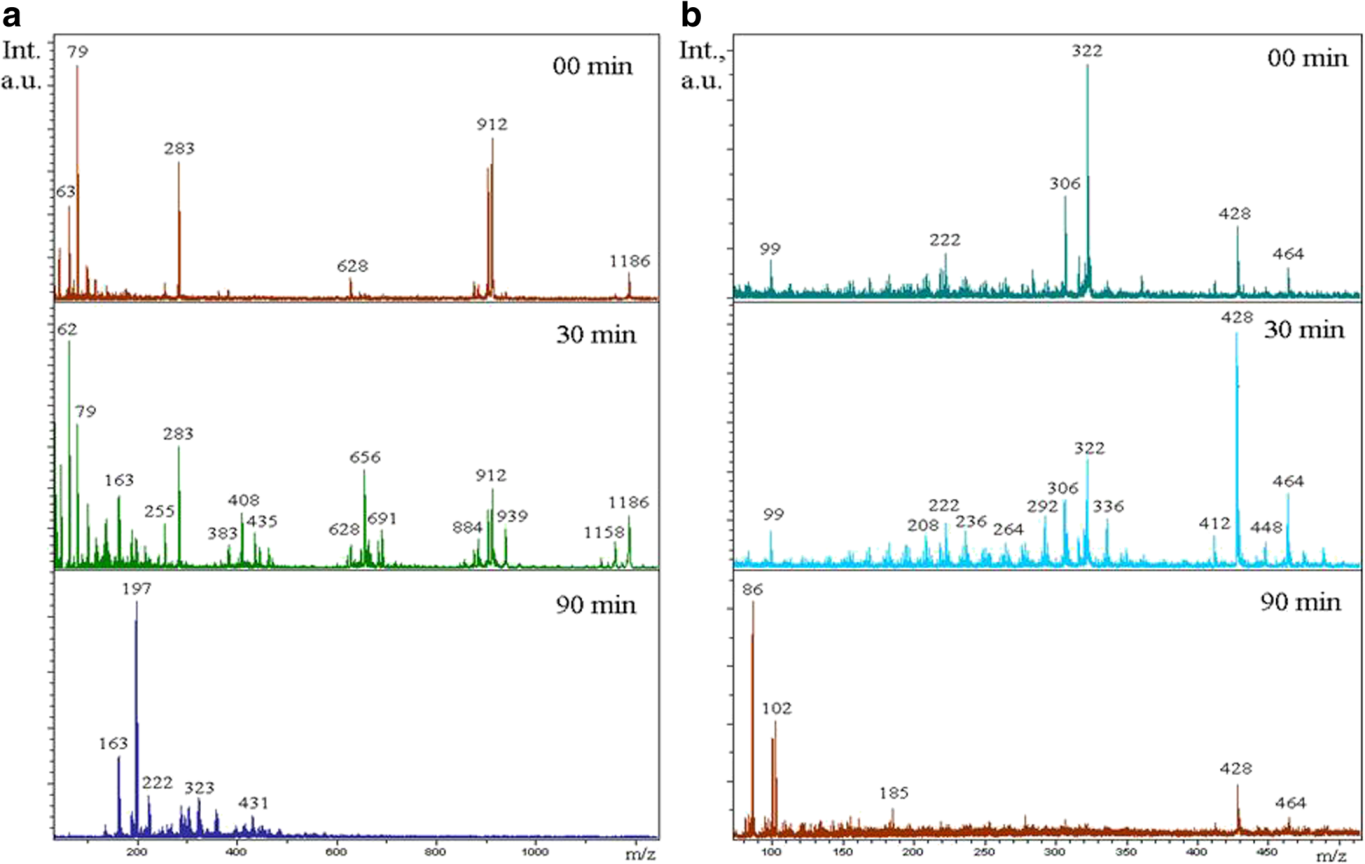
LDI spectra of stearic acid from mesoporous TiO_2_ 400^о^С film after 0-, 15-, and 75-min irradiation. Positive (**a**) and negative (**b**) modes

In the LDI spectra (Fig. 6), before irradiation, the parent SA molecule (m/z = 284) signals are registered in positive mode as cations SA + Na (m/z 306) and SA + K (m/z 322); in negative mode, intensive peaks with m/z 910 and 1184 belonging to SA aggregates were detected. After 15 min of irradiation in positive as well as in negative mode, growths in quantity and intensity of SA peaks were observed. In the field of m/z 428, 656, and 911, 1186 groups of peaks belonging to SA-associated desorption from a superhydrophilic TiO_2_ surface were registered. These results agree with our data about TiO_2_ film wettability. Under UV illumination, all the used films exhibited hydrophilicity with water contact angle of 0°, which gives rise to hydrophobic fat acid molecule self-association and their active desorption from the hydrophilic surface. Then (after 75 min of irradiation), all these peaks are diminished, and intermediate products of SA degradation process with masses m/z = 197, 102, 86, 62, and 35 appear simultaneously with decreasing of peaks of the asymmetric and symmetric C–H stretching modes of the CH_2_ group in the FT-IR spectra.

## Conclusions

Self-cleaning titania photocatalyst films have been synthesized via template-assisted sol–gel method. The films have a surface area of 390–540 m^2^/g with pore size distribution narrowly centered around 10 nm. These films have great potential for applications in LDI spectroscopy as substrates (wafers) with variable hydrophilic properties: they show lower contact angles after UV irradiation than as-prepared films and are more effective in desorption of probe organic (stearic acid) during LDI experiments due to self-association of hydrophobic fat acid molecules.

## Abbreviations

AFM: atomic force microscopy
IR: infrared
LDI-MS: laser desorption–ionization mass spectrometry
SEM: scanning electron microscopy
*S*_sp_: specific surface area
UV light: ultraviolet light
XRD: X-ray diffraction
